# Loeffler endocarditis as a rare cause of heart failure with preserved ejection fraction: A case report and review of literature

**DOI:** 10.1097/MD.0000000000010079

**Published:** 2018-03-16

**Authors:** Ming Gao, Weihua Zhang, Waiou Zhao, Ling Qin, Fei Pei, Yang Zheng

**Affiliations:** Department of Cardiology, The First Hospital of Jilin University, Changchun, China.

**Keywords:** heart involvement, HES, hypereosinophilic syndrome, Löffler endocarditis

## Abstract

**Rationale:**

Hypereosinophilic syndrome (HES) is a rare disease characterized by hypereosinophilia and its ensuing organ damage. Cardiac involvement is divided into 3 chronological stages: an acute necrotic stage; a thrombus formation stage; and a fibrotic stage. Infiltration of the myocardium by eosinophilic cells followed by endomyocardial fibrosis is known as “Loeffler endocarditis.”

**Patient concerns:**

We report a case of a 60-year-old man diagnosed with left-sided restrictive cardiomyopathy.

**Diagnosis:**

The patient experienced heart failure with preserved ejection fraction. The cardiac MRI showed intense, linear, delayed gadolinium enhancement of the endocardium of the lateral wall of the left ventricle, and obliteration of the LV apex. He was ultimately identified as Loeffler endocarditis.

**Intervention:**

A bone marrow smear and biopsy revealed the FIP1L1-PDGFRA fusion gene was positive in 82% of segmented nucleated cells.

**Outcome:**

Our patient responded well to prednisone at 1 mg/kg/d.

**Lessons:**

HES is a rare disease that often afflicts the heart. Cardiac involvement in hypereosinophilia, especially Loeffler endocarditis, carries a poor prognosis and significant mortality. Early detection and treatment of the disease is therefore essential. Further studies are needed to ascertain therapeutic corticosteroid dosages and develop targeted gene therapies, both important steps to ameliorate the effects of Loeffler endocarditis and improve patient outcomes.

## 1 Introduction

Hypereosinophilia is defined by the presence of ≥ 1500/mm^3^ eosinophils in the peripheral blood, and may be reactive, neoplastic, or idiopathic.^[1,2]^ A marked and persistent overproduction of eosinophils that subsequently infiltrate and damage multiple organs via a toxic protein is referred to as hypereosinophilic syndrome (HES). The age-adjusted incident rate of HES is approximately 0.036 per 100,000 person-years.^[3]^ The clinical manifestations of HES are variable and depend on which organ is targeted by the proliferative eosinophils. Cardiac manifestations occur in half of patients with HES and are a major cause of morbidity and mortality among HES patients. Infiltration of the myocardium by eosinophilic cells following endomyocardial fibrosis is known as “Loeffler endocarditis.”^[4]^

In the present study, we report a case of a 60-year-old man diagnosed with left-sided restrictive cardiomyopathy with a preserved ejection fraction, which was identified as Loeffler endocarditis.

## 2 Case report

**Figure 1:**
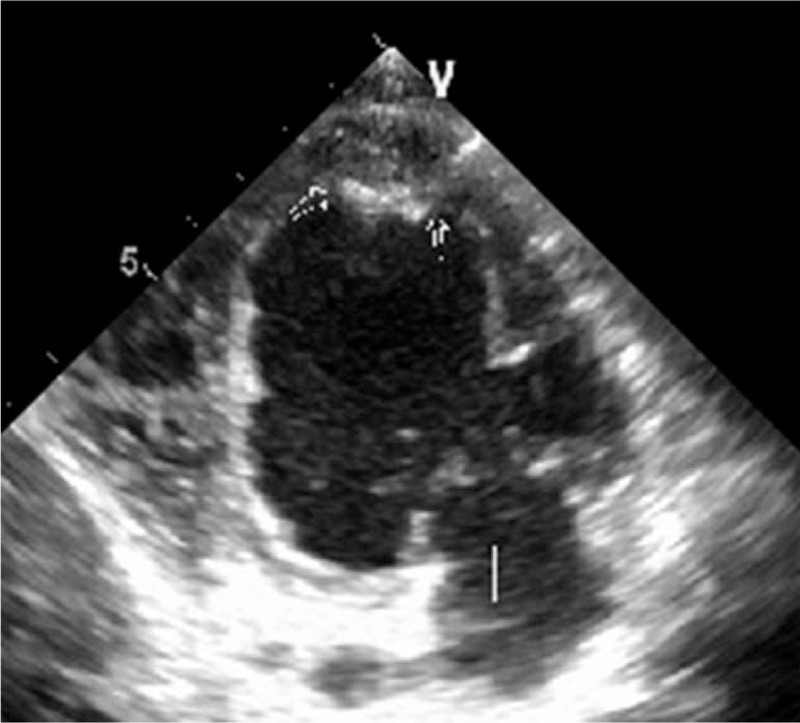
Two-dimensional echocardiography in the apical 4 chamber view showing a mass compatible with a large thrombus on the endocardial surface of the apex of the left ventricle.

**Figure 2:**
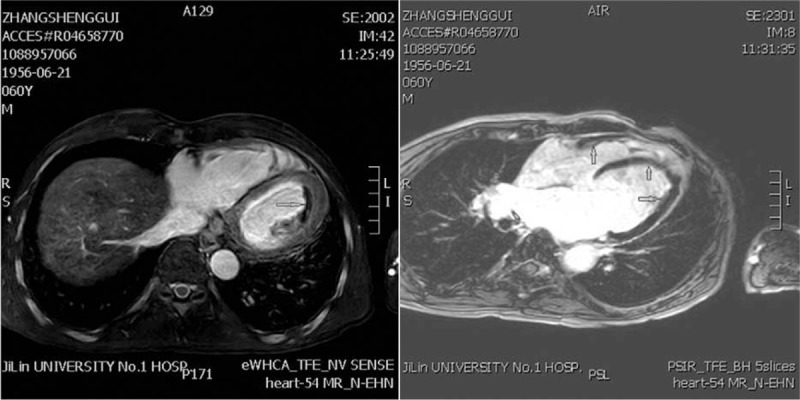
Cardiac magnetic resonance showing late gadolinium enhancement of endocardium of lateral of the left ventricle and obliteration of the LV apex.

**Figure 3:**
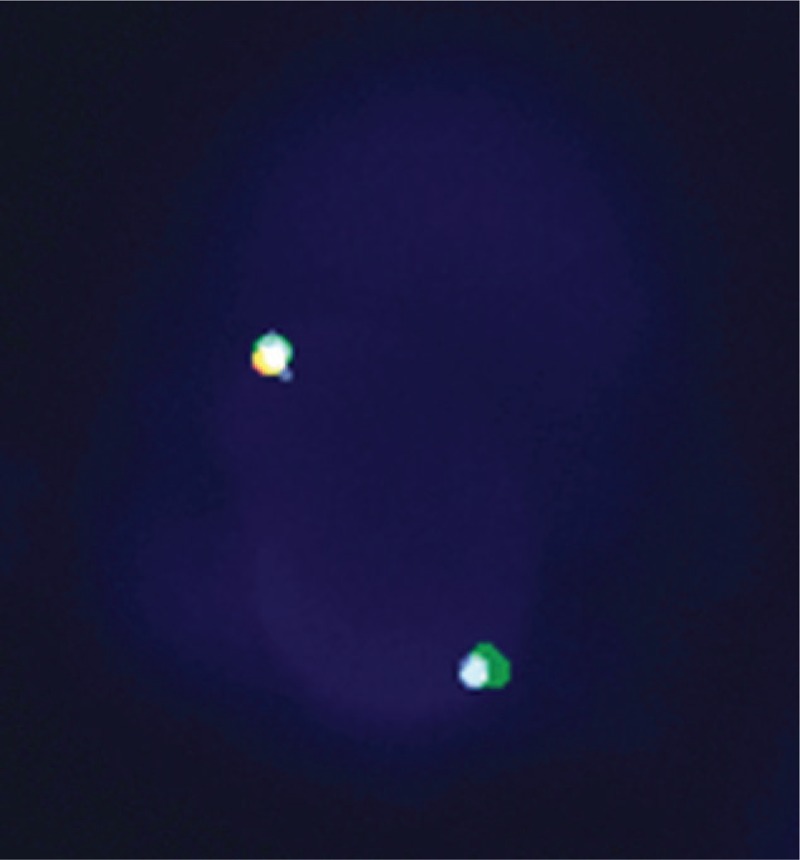
The FIP1L1/PDGFRA fusion gene was positive by FISH analysis in the peripheral blood. The write arrow indicates the FIP1L1/PDGFRA fusion signal in the FISH image. The red arrow indicates the normal signal.

A 60-year-old male with an unremarkable prior medical history presented to our institution with progressively worsening dyspnea on exertion and paroxysmal nocturnal dyspnea for a duration of 20 days. The physical examination was within normal limits, apart from a finding of bilateral crackles of the lung bases. His complete blood count at presentation showed a normal white blood cell count (7280/mL), with 30% neutrophils, 13% lymphocytes, and 5% eosinophils, but demonstrated hypereosinophilia (absolute eosinophil counts 3610/mL, normal 20–520/mL), and severely elevated pro-B-type natriuretic peptides of 3710 pg/mL. Echocardiography revealed an apical left ventricular infiltration with a 14 × 7 mm thrombus that was associated with leaflet restriction and moderate mitral and tricuspid valve regurgitation (Fig. 1). Both atriums were enlarged and a restrictive left ventricle (LV) filling pattern was noted. Both ventricles displayed normal dimensions in the absence of regional wall motion abnormalities, and both ventricular systolic functions were preserved (left ventricular ejection fraction 64%). The estimated systolic pulmonary artery pressure was 71 mm Hg. Electrocardiography (ECG) showed a right axis deviation and T waves changes. Cardiac magnetic resonance imaging (MRI) was performed to aid in the diagnosis. The cardiac MRI showed intense, linear, delayed gadolinium enhancement of the endocardium of the lateral wall of the LV, and obliteration of the LV apex (Fig. 2). A bone marrow smear and biopsy revealed a markedly elevated eosinophil count (>36%), but was absent dysplasia and a number of blasts, and there was no evidence of myeloproliferative disorder. The FIP1L1-PDGFRA fusion gene was positive in 82% of segmented nucleated cells, based on fluorescence in situ hybridization (FISH) analysis (Fig. 3). An additional, extensive workup of secondary causes of eosinophilia proved negative and excluded other causes, leading to the final diagnosis of idiopathic HES with heart involvement. Treatment was started immediately with warfarin and a high dose of prednisone (1 mg/kg/d). Over the next few months, the patient began to feel much better, demonstrating marked improvement in his symptoms, with no complaints of shortness of breath or dyspnea on exertion. Eosinophil counts also normalized.

The study protocol was approved by the ethics review board of the First Hospital of Jilin University (No. 2016–263). Informed consent was obtained from the patient for his information used for research.

## 3 Discussion

Eosinophils are granulocytes that play a role in the immune response to inflammation and infection. In healthy subjects, the upper normal limit of eosinophils in the peripheral blood is 3–5% (absolute eosinophil count of 350–500/mm^3^).^[5,6]^ Extensive infiltration of eosinophils in tissue results in organ damage through associated tissue fibrosis via the secretion of TGF-β and IL-1β.^[7,8]^ A chronic state of eosinophil proliferation and infiltration can lead to a diagnosis of HES.

HES is defined by 1 or more of the followings:

- an elevated eosinophil count (>1500/mm^3^) on 2 separate tests (≥1 mo);
- tissue hypereosinophilia based on >20% eosinophils in a bone marrow section;
- marked disposition of eosinophilic granule proteins in tissue plus organ damage directly due to hypereosinophilia.^[9,10]^

Our patient presented with symptoms of heart failure, and demonstrated a moderately elevated eosinophil count. The diagnosis of HES with cardiac disorder was made by cardiac MRI, the current non-invasive gold standard for diagnosis.

The underlying causes of HES are various and were classified in 2011 at the Working Conference on Eosinophil Disorders and Syndromes into following terms: hereditary (familial) HE variant; primary (clonal/neoplastic) HE variant; secondary (reactive) HE; and HE of undetermined significance/idiopathic variant.^[9]^ Our patient received a complete workup to find the underlying etiologies, and the negative results led to the diagnosis of idiopathic HES.

HES is a potentially fatal disease with cardiac involvement in about 40% to 50% of all cases.^[11]^ Cardiac manifestations, particularly eosinophilic myocarditis and endomyocardial fibrosis, are typical causes of mortality in HES. Eosinophilia cardiac disease was first documented in 1936 by Löffler, who described 2 cases of “endocarditis parietalis fibroplastica,” a condition consistent with the endomyocardial fibrosis seen in HES at later stages of cardiac disease. Cardiac involvement usually follows 3 stages. The first stage, frequently asymptomatic, begins with acute necrosis and eosinophilic infiltration and toxic degranulation. The second (thrombotic) stage is characterized by formation of mural thrombi and thrombus. Both ventricles can be involved, but the thrombus most often occurs at the apex of the LV. The last (fibrotic) stage occurs when the granulation tissue is replaced by fibrosis and restrictive cardiomyopathy ensues. Severe valvular disease is a common complication during this stage, and patients present with clinical restrictive features but preserved systolic function.^[10]^ It is important to note that the stages may overlap^[12]^; the degree of cardiac dysfunction does not necessarily correlate with the degree of eosinophilia.

Histological evidence is necessary for the diagnosis of HES; however, the invasive endocardial biopsy procedure necessary to obtain sample is difficult to implement clinically. It may be more valuable in diagnosing early cardiac involvement, but the acquisition of an adequate biopsy sample is difficult, especially in the presence of endocardial fibrosis and superimposed thrombi.^[13]^ By contrast, echocardiography is a very useful first-line method to detect impairment of cardiac and valvular function, ant to visualize thrombus and pericardial effusions and their dynamics. Echocardiography should be used in cases of moderate (1500/mm^3^) to severe eosinophilia (5000/mm^3^),^[6]^ whereas the initial necrotic stage may be undetectable by echocardiography. Cardiac MRI is currently the gold standard for non-invasive diagnosis, monitoring, and prognostic stratification of myocarditis. It can easily detect endocardial fibrosis with subendocardial late gadolinium enhancement before functional consequences, and reduce the need for invasive biopsy in HES patients. Genetic testing for Fip1-like1-platelet-derived growth factor receptor (alpha) fusion gene (FIP1L1-PDGRFA) has recently been reported in a few cases of HES and has been added to the diagnostic algorithm of HES.^[14]^ The FP fusion gene can activate tyrosine kinase activity, stimulate proliferation, and mediate survival of eosinophils in HES.^[14]^ The prevalence of FIP1L1-PDGRFA fusion is 10% or less in idiopathic HES.^[2,15]^ Presence of the mutation appears to correlate with more common cardiac involvement.^[16]^ The tyrosine kinase inhibitor imatinib is a specific inhibitor of the PDGFR alpha receptor and has been a clear target in the development of treatments for the FLP1L1-PDGFRA mutation. Imatinib can achieve complete hematological and molecular remission within weeks to months,^[16,17]^ but cannot eliminate the FLP1L1-PDGFRA clone in most patients.^[18]^

Therapeutic interventions for HES typically proceed in a stepwise fashion, beginning with immunosuppressive treatment corticosteroids (CS) to reduce eosinophil count and counteract inflammation.^[19]^ There is little consensus regarding the initial dosage of CS or the treatment duration. In a multicenter study of 188 subjects with HES, 179 of whom had the FLP1L1-PDGFRA mutation and were treated with CS, 65% were non-responders.^[20,21]^ Among the responders, there are no clear evidence-based recommendations that can be given. It seems reasonable to adjust the dosages of CS and the treatment duration with respect to the severity of EM manifestation. Recommended starting doses are typically 0.5 to 1 mg prednisone/kg body weight. The prednisone dose required to maintain disease control is highly variable from 1 patient to another overtime. Our patient, who tested positive for the FLP1L1-PDGFRA mutation responded well to prednisone at 1 mg/kg/d.

## 4 Conclusion

HES is a rare disease that often afflicts the heart. Cardiac involvement in hypereosinophilia, especially Loeffler endocarditis, carries a poor prognosis and significant mortality. Early detection and treatment of the disease is therefore essential. Further studies are needed to ascertain therapeutic corticosteroid dosages and develop targeted gene therapies, both important steps to ameliorate the effects of Loeffler endocarditis and improve patient outcomes.

## References

[1] Gotlib J World Health Organization-defined eosinophilic disorders: 2015 update on diagnosis, risk stratification, and management. Am J Hematol 2015;90:1077–89. 26486351 10.1002/ajh.24196

[2] Gotlib J World Health Organization-defined eosinophilic disorders: 2017 update on diagnosis, risk stratification, and management. Am J Hematol 2017;92:1243–59. 29044676 10.1002/ajh.24880

[3] Crane MM Chang CM Kobayashi MG Incidence of myeloproliferative hypereosinophilic syndrome in the United States and an estimate of all hypereosinophilic syndrome incidence. J Aller Clin Immunol 2010;126:179–81. 10.1016/j.jaci.2010.03.035 PMC5781228 20639012

[4] Tai PC Ackerman SJ Spry CJ Deposits of eosinophil granule proteins in cardiac tissues of patients with eosinophilic endomyocardial disease. Lancet (London, England) 1987;1:643–7. 10.1016/s0140-6736(87)90412-0 2882081

[5] Al Ali AM Straatman LP Allard MF Eosinophilic myocarditis: case series and review of literature. Can J Cardiol 2006;22:1233–7. 17151774 10.1016/s0828-282x(06)70965-5 PMC2569073

[6] Rothenberg ME Eosinophilia. N Engl J Med 1998;338:1592–600. 9603798 10.1056/NEJM199805283382206

[7] Gomes I Mathur SK Espenshade BM Eosinophil-fibroblast interactions induce fibroblast IL-6 secretion and extracellular matrix gene expression: implications in fibrogenesis. J Aller Clin Immunol 2005;116:796–804. 10.1016/j.jaci.2005.06.031 16210053

[8] Noguchi H Kephart GM Colby TV Tissue eosinophilia and eosinophil degranulation in syndromes associated with fibrosis. Am J Pathol 1992;140:521–8. 1739138 PMC1886427

[9] Valent P Klion AD Horny HP Contemporary consensus proposal on criteria and classification of eosinophilic disorders and related syndromes. J Aller Clin Immunol 2012;130: 607–12 e9. 10.1016/j.jaci.2012.02.019 PMC4091810 22460074

[10] Mankad R Bonnichsen C Mankad S Hypereosinophilic syndrome: cardiac diagnosis and management. Heart (British Cardiac Society) 2016;102:100–6. 26567231 10.1136/heartjnl-2015-307959

[11] Ogbogu PU Rosing DR Horne MK 3rd Cardiovascular manifestations of hypereosinophilic syndromes. Immunol Aller Clin North Am 2007;27:457–75. 10.1016/j.iac.2007.07.001 PMC2048688 17868859

[12] Weller PF Bubley GJ The idiopathic hypereosinophilic syndrome. Blood 1994;83:2759–79. 8180373

[13] Cunningham KS Veinot JP Butany J An approach to endomyocardial biopsy interpretation. J Clin Pathol 2006;59:121–9. 16443725 10.1136/jcp.2005.026443 PMC1860308

[14] Cogan E Roufosse F Clinical management of the hypereosinophilic syndromes. Expert Rev Hematol 2012;5:275–89. quiz 90. 22780208 10.1586/ehm.12.14

[15] Pardanani A Brockman SR Paternoster SF FIP1L1-PDGFRA fusion: prevalence and clinicopathologic correlates in 89 consecutive patients with moderate to severe eosinophilia. Blood 2004;104:3038–45. 15284118 10.1182/blood-2004-03-0787

[16] Klion AD Noel P Akin C Elevated serum tryptase levels identify a subset of patients with a myeloproliferative variant of idiopathic hypereosinophilic syndrome associated with tissue fibrosis, poor prognosis, and imatinib responsiveness. Blood 2003;101:4660–6. 12676775 10.1182/blood-2003-01-0006

[17] Tefferi A Patnaik MM Pardanani A Eosinophilia: secondary, clonal and idiopathic. Br J Haematol 2006;133:468–92. 16681635 10.1111/j.1365-2141.2006.06038.x

[18] Akuthota P Weller PF Spectrum of eosinophilic end-organ manifestations. Immunol Aller Clin North Am 2015;35:403–11. 10.1016/j.iac.2015.04.002 PMC4515759 26209892

[19] Helbig G Wisniewska-Piaty K Francuz T Diversity of clinical manifestations and response to corticosteroids for idiopathic hypereosinophilic syndrome: retrospective study in 33 patients. Leuk Lymphoma 2013;54:807–11. 22988896 10.3109/10428194.2012.731602

[20] Khoury P Abiodun AO Holland-Thomas N Hypereosinophilic syndrome subtype predicts responsiveness to glucocorticoids. J Aller Clin Immunol Pract 2017. 10.1016/j.jaip.2017.06.006 PMC5760470 28757367

[21] Ogbogu PU Bochner BS Butterfield JH Hypereosinophilic syndrome: a multicenter, retrospective analysis of clinical characteristics and response to therapy. J Aller Clin Immunol 2009;124: 1319–25 e3. 10.1016/j.jaci.2009.09.022 PMC2829669 19910029

